# Narrative Coherence and Identity: Associations With Psychological Well-Being and Internalizing Symptoms

**DOI:** 10.3389/fpsyg.2019.01171

**Published:** 2019-05-24

**Authors:** Louise Vanden Poel, Dirk Hermans

**Affiliations:** Centre for the Psychology of Learning and Experimental Psychopathology, Faculty of Psychology and Educational Sciences, KU Leuven, Leuven, Belgium

**Keywords:** autobiographical memory, narrative coherence, memory coherence, identity, self

## Abstract

There are considerable differences in the ways in which individuals remember and try to make meaning out of past personal experiences. One autobiographical memory characteristic that has been receiving growing attention is narrative coherence, or the extent to which an individual is able to construct coherent accounts of their autobiographical memories. Previously, differences in narrative coherence have been found to be related to well-being, with more coherent individuals displaying higher levels of well-being. This study aimed to extend previous findings by examining the associations between narrative coherence, identity functioning, psychological well-being, and internalizing symptoms in a sample of 395 American young adults (ages 18**–**30). We examined whether these associations differed within individuals as a function of the identity-relevance of the memory. In line with our prediction, narrative coherence was positively related to healthy identity functioning. However, the hypothesis that narrative coherence would be positively related to psychological well-being and negatively related to internalizing symptoms was only partially confirmed (i.e. only for personal growth and anxiety symptoms). These findings indicate that the relation between coherence and well-being might be more complex than originally assumed. Contrary to our predictions, it was not significantly more beneficial for an individual to narrate about a memory with high identity-relevance compared to a memory with low identity-relevance. Thus, it might be the individual’s general ability to include identity content within their narratives of personal experiences that moderates the association between coherence and well-being, rather than the identity-relevance of the specific memory. In the current study, we also found a significant gender effect, with women being more coherent than men. Furthermore, exploratory analyses indicated that being more coherence was associated with fewer internalizing symptoms and fewer identity-related struggles in men, but not in women. Possible explanations for the observed gender differences are discussed. Future longitudinal and experimental studies are needed to further clarify the link between narrative coherence, identity, and well-being.

## Introduction

Autobiographical remembering serves important functions for the self, including being able to effectively guide our future behavior, as well as to form and strengthen social bonds (Bluck and Alea, 2002). Furthermore, interpreting and linking past events enables us to construct a personal identity and maintain a stable sense of self (Wilson and Ross, 2003). Although remembering and trying to make meaning out of past personal experiences is a distinctive and universal part of being human, vast individual differences in autobiographical memory characteristics can be observed (e.g., Bauer et al., 2003; Nelson and Fivush, 2004). Importantly, previous research shows that such individual differences in how personal experiences are recalled and narrated about can be linked to certain types of psychopathology. For example, depressed individuals tend to recall their autobiographical memories in a more overgeneral and less specific manner than healthy controls (Williams et al., 2007). Another characteristic of autobiographical memories that has been receiving growing interest is narrative coherence, or the extent to which one can construct coherent accounts of personally experienced events.

Whereas there are several conceptualizations of narrative coherence throughout different domains in the literature, we focus on the model of Reese et al. (2011), in which coherence is regarded as a multidimensional construct. According to the authors, there are three dimensions of narrative coherence that develop independently and at a different rate across the lifespan. For a narrative to be coherent, it must first contain specific information regarding the time and location of the event (context). Second, the order in which different actions of the event took place is clear or can be inferred through the use of temporal indications within the narrative (chronology). Finally, the narrative includes personal evaluations and affective utterances, and revolves around a clear topic that is introduced, elaborated upon, and eventually resolved (theme). Research has shown that there is at least some stability regarding individual differences in narrative coherence (McLean et al., 2017; Waters et al., 2019).

Narrating about personal experiences in a coherent manner is assumed to be beneficial for one’s well-being, as this implies that the individual has been able to make meaning out of that particular experience (Boals et al., 2011). In contrast, narrating in a very incoherent manner is thought to be related to psychopathology. Accordingly, the coherence of young adults’ autobiographical narratives of personally significant events has been found to be positively associated with psychological well-being, with the experience of purpose and meaning in life, and with maintaining positive relationships (Waters and Fivush, 2015), and negatively associated with depressive symptoms (Vanderveren et al., 2019). In children, higher coherence has also found to be associated with less internalizing and externalizing problems (von Klitzing et al., 2000; Müller et al., 2014). Moreover, some studies showed that high narrative coherence can buffer the negative impact of adverse life experiences and family risk factors (Müller et al., 2014; Stadelmann et al., 2015).

Notwithstanding these promising findings, there are still inconsistencies in the literature, as some studies do not find a significant association between narrative coherence and well-being (Fivush et al., 2003). There is evidence that this association might be influenced by event-specific characteristics of the memory that is narrated about (Waters et al., 2019). For example, in a study of Waters and Fivush (2015), well-being has been found to be positively related to the coherence of narratives about unique events but unrelated to the coherence of narratives about recurring events. In the same study, the authors also examined the importance of the memory’s relevance to identity, by coding the extent to which participants’ narratives of personal experiences included identity content (using the Self-Function Scale; Waters et al., 2014). Because each participant wrote about two unique events, the identity scores were summed across the two events, in order to obtain a total identity content score for each participant. Interestingly, Waters and Fivush (2015) found an interaction effect between narrative coherence and identity. More specifically, participants who narrated in a very coherent manner reported higher levels of well-being whereas participants who narrated in a very incoherent manner reported lower levels of well-being, but only if their narratives strongly focused on identity (e.g., if they elaborated on how the event had an impact on their personal goals or attitudes about the self). When participants’ narratives had no explicit focus on identity, coherence and well-being were not related. Thus, the relevance to identity seems to moderate the relationship between narrative coherence and well-being.

Although the results of Waters and Fivush (2015) are definitely noteworthy, they are also somewhat ambiguous. It is unclear whether the moderating effect of identity-relevance is best accounted for by individual differences in narrating style or by specific characteristics of the event that is being narrated about. As already noted, there are stable individual differences in narrative coherence, with some individuals being generally more coherent than others when narrating about personal experiences (Waters et al., 2019). In a similar way, individuals are likely to differ in the extent to which they tend to include identity content within their narratives. However, characteristics of the chosen events might have also influenced the identity scores, because some events are in itself more relevant to one’s identity or sense of self than others. In their paper, Waters and Fivush (2015) do not really differentiate between these two possibilities (individual characteristics versus event-specific characteristics). While their operationalization of identity-relevance (calculating a total score for each participant) relates to individual differences in the ability to include identity content within narratives, they also make claims about the impact of the events’ identity-relevance: “Further, we found that this relation [between narrative coherence and psychological well-being] was moderated by the narratives’ relevance to identity …” (p. 441). One of the aims of the current study was to further clarify if there is an effect of the narrative’s relevance to identity on the association between participants’ narrative coherence and their well-being, while controlling for individual differences in narrating style. To do so, we manipulated identity-relevance within participants.

Thus far, most research linking identity and autobiographical narratives stems from the life story literature, which studies the overarching life narrative that individuals construct from single episodic memories, relating themselves through past, present, and future (Singer, 2004; McAdams and McLean, 2013). This narrative identity emerges in adolescence and provides individuals with a sense of continuity and purpose in life (Habermas and Bluck, 2000; Habermas, 2019). Akin to narrative coherence of single episodic memories, the overall coherence of the life story has been found to be predictive of the individual’s psychological functioning (Adler et al., 2016). In a study of Baerger and McAdams (1999), the coherence of one’s life story was negatively associated with self-reported symptoms of depression. Concerning identity, individuals whose identity is disturbed (e.g., individuals with symptoms or a diagnosis of Borderline Personality Disorder), tend to have life narratives that are significantly more incoherent than healthy controls (Adler et al., 2012; Jørgensen et al., 2012; Lind et al., 2018). Whereas this research is concerned with the coherence of the life story (global coherence), the findings from Waters and Fivush (2015) seem to suggest that identity is also related to the coherence of single episodic memories (local coherence).

The aim of the current study was to examine the link between narrative coherence of personally significant events and identity functioning in a sample of young adults. In addition, we aimed to further examine the associations between narrative coherence, psychological well-being, and internalizing symptoms. We predicted that coherence would be positively correlated with healthy identity functioning and psychological well-being, and negatively correlated with identity-related struggles and internalizing symptoms. Furthermore, we also wanted to examine whether these associations differed depending on the identity-relevance of the narrated event, by explicitly instructing all participants to write about one memory with high identity-relevance and one memory with low identity-relevance. Unlike Waters and Fivush (2015), we manipulated identity-relevance beforehand and compared the associations between coherence of the collected narratives and well-being within participants. Because both the narrative about the memory with high identity-relevance and the narrative about the memory with low identity-relevance come from the same individual, any confounding effects of the individuals’ general narrating style are controlled for. We predicted that within individuals, coherence for memories with high identity-relevance would correlate more strongly with the variables of interest, than would be the case for memories with low identity-relevance. In other words, we expected that it would be especially beneficial for an individual to coherently narrate about memories with high relevance to identity or sense of self.

## Materials and Methods

### Participants

A sample of 395 American young adults (ages 18–30, *M* = 25.66, SD = 3.00, 274 females) was recruited via *Amazon’s Mechanical Turk* (MTurk). Only MTurk workers that met the predetermined requirements (age 18–30 and located in US) could participate and received a compensation of $4. Nearly half of the participants (49%) reported having completed a college or graduate school degree.

### Procedure

Eligible MTurk workers were directed to an online survey in Qualtrics. After providing informed consent, all participants were asked to write about two personally significant memories: one with high identity-relevance and one with low identity-relevance. The order of the writing task was counterbalanced across participants. Subsequently, they rated each memory on several characteristics: the intensity with which they experienced various emotions (both negative and positive) during writing, how vivid they recalled the memory, and how important they considered the memory to be (on 7-point Likert scales ranging from *not at all* to *extremely*). Finally, all participants filled out questionnaires concerning their identity functioning, psychological well-being, and experienced internalizing symptoms. On average, participants took 57 min to complete the survey. Participants were naïve to the aim of the study. This study was carried out in accordance with the recommendations of the Declaration of Helsinki and the European General Data Protection Regulation (GDPR). The study protocol was approved by the Social and Societal Ethics Committee of the KU Leuven and pre-registered on AsPredicted.^1^ All subjects gave written informed consent in accordance with the Declaration of Helsinki.

### Materials

*Writing Task.* Participants were asked to write about a memory with high relation to identity and one with little to no relation to identity, using the following instructions:

I would like to ask you to recall and write about a memory of an event with [high or little to no] relation to your identity or sense of self. This can be a recent event, or something that happened years ago. The memory can be highly positive or negative, or both, in how it makes you feel, but it should have had a significant impact on your life. As you write about the event, you can describe the facts, as well as the thoughts and feelings that went with it. Try to be as specific and detailed as possible, there is no time limit for this exercise.

Because it was crucial that participants could get a good understanding of what was meant by high and low identity-relevance, they were provided with a detailed description of both before the writing task. Additionally, they were given some illustrative examples as well as questions on this information, which they needed to answer correctly to continue with the actual task. A memory with high identity-relevance was described as a memory about an event that (1) reflects (or led to changes in) your personal goals and/or; (2) contains information about (or led to changes in) how you see yourself as a person. A memory with low identity-relevance is a memory about an event that, although impactful, (1) does not reflect any of your personal goals and (2) does not contain any information about how you see yourself as a person. This conceptualization of identity-relevance was based on the Self-function coding scheme of Waters et al. (2014), that has been previously used to code for identity content within personal narratives (Waters and Fivush, 2015). It was stressed that the extent to which a certain memory is related to identity is subjective: that is, a similar event (e.g., high school graduation) might be highly related to identity for one person, whereas for another person this event might have no particular relation to their identity. Participants had to write a minimum of 1,000 characters (approximately 12 lines) on each event before they could continue, to make sure that they would put effort in the writing task and not just skip ahead to the next part of the survey. Example narratives are presented in Appendix A.

*Narrative Coherence.* All collected narratives were coded for coherence using the Narrative Coherence Coding Scheme (Reese et al., 2011). The three dimensions of context, chronology, and theme were scored separately along a 4-point scale and summation of the three scores yielded a total score for coherence. Participants’ overall coherence was calculated by averaging their scores across both memories. Two independent coders established good interrater reliability for all dimensions (*κ* = 0.75 for context, 0.73 for chronology, and 0.80 for theme) on a subset of 160 narratives (20%). Disagreements were solved through face-to-face discussion. Hereafter, the main researcher independently coded the remaining narratives (80%).

*Identity Functioning.* The Self-Concept and Identity Measure (Kaufman et al., 2015), which consists of three subscales, was used to assess participants’ identity functioning. The Consolidated Identity subscale consists of items capturing the individual’s experienced sense of consistency over time (e.g., “I always have a good sense about what is important to me”) and is thought to reflect healthy identity functioning. In contrast, the other two subscales measure maladaptive identity functioning, with Disturbed Identity capturing a variety of identity-related struggles (e.g., “I have never really known what I believe or value”) and Lack of Identity capturing more clinical levels of identity problems (e.g., “I no longer know I am”). Summation of all subscales yields a total score (range: 27–189), with higher values reflecting greater problems in identity functioning. For this study, the SCIM showed excellent overall internal consistency (*α* = 0.92), and at least good internal consistency for all subscales (*α* = 0.80, 0.86, and 0.91 for Consolidated Identity, Disturbed Identity, and Lack of Identity, respectively).

*Psychological Well-Being.* Participants’ psychological well-being was assessed by the Ryff’s Psychological Well-Being Scales (PWB; Ryff and Keyes, 1995), which measures well-being across six theoretically-guided dimensions: autonomy (e.g., “My decisions are not usually influenced by what everyone else is doing”), positive relationships (e.g., “I feel like I get a lot out of my friendships”), environmental mastery (e.g., “I am quite good at managing the many responsibilities of my daily life”), personal growth (e.g., “I have the sense that I have developed a lot as a person over time”), purpose in life (e.g., “I am an active person in carrying out the plans I set for myself”), and self-acceptance (e.g., “I like most aspects of my personality”). The current study used the 54-item version of the PWB, which showed excellent overall consistency (*α* = 0.96) and good levels of internal consistency for all subscales (*α* = 0.80–0.90). Items are equally divided over the subscales and the total score (range: 54–324) is an indication of participants’ general psychological well-being, with higher scores reflecting higher well-being.

*Internalizing Symptoms.* Participants’ experienced internalizing symptoms were measured by the Depression, Anxiety, and Stress Scale (DASS; Lovibond and Lovibond, 1995). The DASS consists of three subscales and is able to successfully discriminate between symptoms of depression (e.g., “I felt that I had nothing to look forward to”), anxiety (e.g., “I felt I was close to panic”), and stress (e.g., “I found it difficult to relax”). Participants had to rate the extent to which they experienced every symptom during the past week (total range: 0–63). This study used the 21-item version (DASS-21) was used, which has shown to have adequate validity and reliability (Henry and Crawford, 2005). In the current study, all subscales showed good to excellent levels of internal consistency (*α* = 0.94, 0.88, and 0.89 for the Depression, Anxiety, and Stress scale, respectively).

### Data Analysis

The hypothesis that coherence would be associated with identity functioning, psychological well-being, and internalizing symptoms was tested by calculating Pearson correlation coefficients between these variables. Because we observed a significant gender difference in coherence, we repeated these analyses for women and men separately. To test whether the associations would be significantly stronger for memories with high identity-relevance compared to memories with low identity-relevance, we conducted Steiger’s *Z*-tests (using a web-based calculator of Hoerger, 2013).

## Results

### Descriptive Statistics

Descriptive statistics and correlations of the self-report measures of the current sample are presented in Table 1. Analysis of participants’ ratings revealed significant differences between the two types of memories. Memories with high identity-relevance led to more intense emotions (both positive and negative) during recall, *t*(394) = 11.29, *p* < 0.001, *d* = 0.57, were recalled more vividly, *t*(394) = 7.73, *p* < 0.001, *d* = 0.39, and were considered to be more important, *t*(394) = 17.14, *p* < 0.001, *d* = 0.86, than memories with low identity-relevance. Also, memories with high identity-relevance were significantly more coherent than memories with low identity-relevance, *t*(394) = 5.93, *p* < 0.001, *d* = 0.30, and there was a moderate correlation between the coherence scores of both memory types, *r*(393) = 0.32, *p* < 0.001. Across participants, there was a significant effect of gender, *t*(393) = −3.73, *p* = 0.001, *d* = 0.41, with women being more coherent than men. However, there was no significant difference of education level (high or low) on coherence, *t*(393) = −0.11, *p* = 0.92, *d* = 0.01, and no significant association between narrative coherence and age, *r*(393) = 0.03, *p* = 0.55.

**Table 1:** Descriptive statistics and correlations between the self-report measures.

Measure	*M*	*SD*	Range	Correlation	
				PWB	DASS
PWB	224.07	43.35	123–320	–	
DASS	16.12	14.26	0–62	−0.63**	—
SCIM	75.25	25.25	27–144	−0.77**	0.59**

*N* = 395; PWB = Psychological Well-Being Scale; DASS = Depression, Anxiety, and Stress Scale; SCIM = Self-Concept and Identity Measure.

** *p* < 0.01.

### Associations Between Coherence, Identity, Well-Being, and Internalizing Symptoms

Correlational analysis showed no significant associations between participants’ overall coherence and their psychological well-being, *r*(393) = 0.06, *p* = 0.21, or internalizing symptoms, *r*(393) = −0.09, *p* = 0.06. However, there was a significant negative association between coherence and identity-related problems, *r*(393) = −0.11, *p* = 0.03, indicating that lower coherence was related to more problematic identity functioning. Associations between narrative coherence and the separate subscales of each measure are shown in Table 2.

**Table 2:** Bivariate correlations between narrative coherence and subscales of the self-report measures.

	Narrative coherence	Narrative coherence (women)	Narrative coherence (men)
Autonomy (PWB)	−0.01	0.03	−0.01
Positive relationships (PWB)	0.07	0.04	0.11
Environmental mastery (PWB)	0.05	0.04	0.10
Personal growth (PWB)	0.11*	0.09	0.09
Purpose in life (PWB)	0.08	0.05	0.09
Self-acceptance (PWB)	0.01	0.01	0.05
Depression (DASS)	−0.06	0.00	−0.22*
Anxiety (DASS)	−0.14**	−0.11	−0.22*
Stress (DASS)	−0.06	−0.02	−0.18*
Consolidated identity (SCIM)	0.12*	0.11	0.13
Disturbed identity (SCIM)	−0.09	−0.02	−0.21*
Lack of identity (SCIM)	−0.07	−0.03	−0.19*

*N* = 395, *N* women = 274, *N* men = 121; PWB = Psychological Well-Being Scale; DASS = Depression, Anxiety, and Stress Scale; SCIM = Self-Concept and Identity Measure.

* *p* < 0.05;

** *p* < 0.01.

### Gender Differences in Associations

**Figure 1:**
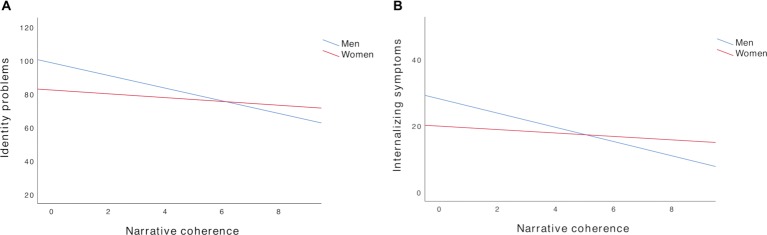
Gender differences in **(A)** identity problems and **(B)** internalizing symptoms as a function of narrative coherence.

Because of the significant gender difference in coherence in this sample, we ran some additional exploratory analyses (not pre-registered) to examine whether the associations between coherence and the self-report measures differed across gender. In women, the associations between coherence and well-being, *r*(272) = 0.05, *p* = 0.40, internalizing symptoms, *r*(272) = −0.04, *p* = 0.47, and identity-related problems, *r*(272) = −0.06, *p* = 0.37, were all non-significant. In men, there also was no significant association between coherence and well-being, *r*(119) = 0.09, *p* = 0.33. However, men’s coherence was negatively correlated with both internalizing symptoms, *r*(119) = −0.22, *p* = 0.01, and identity problems, *r*(119) = −0.21, *p* = 0.02. Thus, for men, higher coherence was associated with fewer symptoms of depression, anxiety, and stress, as well as less problematic identity functioning (Figure 1). Table 2 shows the associations between coherence of both men and women with the subscales of each measure.

### Effect of Identity-Relevance

Next, we wanted to examine whether participants’ coherence scores for memories with high identity-relevance and memories with low identity-relevance would show different associations with the self-report measures. As already indicated, the two coherence scores were moderately correlated, *r*(393) = 0.32, *p* < 0.001. For memories with low identity-relevance, there were no significant associations between coherence and identity-related struggles, *r*(393) = −0.08, *p* = 0.11, psychological well-being, *r*(393) = 0.02, *p* = 0.68, or internalizing symptoms, *r*(393) = −0.09, *p* = 0.07. For memories with high identity-relevance, there were no significant associations between coherence and psychological well-being, *r*(393) = 0.08, *p* = 0.10, or internalizing symptoms, *r*(393) = −0.06, *p* = 0.25, but there was a significant negative association between coherence and identity-related struggles, *r*(393) = −0.10, *p* = 0.045. However, when comparing the strength of these associations, Steiger *Z*-tests revealed no significant differences for either well-being, *Z*_h_ = 1.05, *p* = 0.29, internalizing symptoms, *Z*_h_ = 0.58, *p* = 0.56, or identity functioning, *Z*_h_ = −0.36, *p* = 0.72. Thus, the coherence for memories with high identity-relevance was not significantly more correlated with the variables of interest than the coherence for memories with low identity-relevance. Repeating these analyses separately for men and women did not significantly affect the results.

## Discussion

This study examined the link between narrative coherence, identity, psychological well-being, and internalizing symptoms, while also accounting for the identity-relevance of the narrative. We predicted a positive association between coherence and psychological well-being, and a negative association between coherence and internalizing symptoms. Our hypotheses regarding the associations between coherence and the self-report measures were only partially confirmed. Opposite to previous findings (Müller et al., 2014; Waters and Fivush, 2015), coherence was not significantly related to overall well-being or internalizing symptoms. However, there were significant associations in the expected direction at subscale level. For psychological well-being, higher coherence was associated with more personal growth. Our results suggest that coherence is only beneficial for very specific components of psychological well-being. For internalizing symptoms, we found higher coherence to be significantly associated with fewer anxiety symptoms. Opposite results, where narrative coherence was negatively associated with depressive symptoms but not anxiety symptoms, have also been reported (Vanderveren et al., 2019). Overall, these findings indicate that the relation between coherence and well-being is probably more complex than originally assumed. Other mechanisms (e.g., meaning-making or metacognitive capacity; Dimaggio and Semerari, 2001; Dimaggio et al., 2010; Boals et al., 2011) are necessary in order to gain a better understanding of their relationship. However, because all observed associations were in the expected direction (albeit not significant), we could at least state that more narrative coherence is not detrimental for well-being. Future research could further examine which components of well-being are related to coherence and also focus on possible mechanisms underlying this association.

To the best of our knowledge, the current study is the first to examine the direct association between narrative coherence of single episodic memories (local coherence) and identity. In line with our prediction, higher narrative coherence was positively related to adaptive identity functioning. Thus, individuals who narrate about personal experiences in a highly coherent manner also experience a higher sense of consistency over time. This finding is in line with results from the life story literature, in which narrative coherence of the life story (global coherence) has been found to be impaired in individuals with disturbed identity functioning (Adler et al., 2012). The finding that this association is also present when it comes to narratives of unique personally significant events is interesting in itself. Of course, more research is needed to gain insight in the direction of this relationship. Are coherent autobiographical memories a prerequisite to come to a clear sense of self, or vice versa? In order to clarify this relationship, longitudinal and experimental studies are needed.

Following earlier findings of Waters and Fivush (2015), we hypothesized that it would be especially beneficial for individuals to narrate coherently about memories with a high relation to identity or sense of self. Contrary to our predictions, associations between coherence of memories with high identity-relevance and identity, well-being, and internalizing symptoms, were not significantly stronger compared to the associations with coherence of memories with low identity-relevance. Thus, our findings seem to suggest that it might be the individual’s general ability to include identity content within their narratives of personal experiences that moderates the association between narrative coherence and well-being, rather than the identity-relevance of the specific memory. We do note that the current study just instructed participants to write about events with high relevance to identity, without specifying whether this identity content should be positive or negative in nature. It is possible that the expected associations with coherence are only present if the individual describes positive identity content but not when this content is more negative in nature (Banks and Salmon, 2013). This possibility, as well as other event-specific characteristics of the narrated memory that could have an impact on narrative coherence and its association with well-being, could be further explored in future studies.

Finally, significant gender differences in narrative coherence were observed, with women being more coherent than men. Several studies report gender differences in autobiographical memory characteristics, generally indicating that women are more elaborative and more coherent when narrating their memories (Fivush et al., 2012; Grysman and Hudson, 2013). However, our study showed that higher coherence seems to be especially beneficial for men, in terms of fewer experienced internalizing symptoms and fewer identity-related struggles. In women, we did not find significant associations between coherence and either identity-functioning, well-being, or internalizing symptoms. Although these results have to be interpreted with caution, as we did not formulate prior hypotheses concerning gender differences, previous studies have reported similar findings. More internal state language within personal narratives has been found to be positively associated with well-being in adolescent males, but not in adolescent females (Bohanek and Fivush, 2010). Similarly, repeatedly writing about traumatic and emotional events has been found to be more effective in improving well-being for men than for women (Smyth, 1998; Klein and Boals, 2001; Frattaroli, 2006). Future research could examine whether these gender differences regarding coherence and well-being are replicable and investigate possible mechanisms that could explain why men seem to benefit more from writing in a coherent manner than women. One possible explanation is that men normally tend to engage less in emotional disclosure as a result of gender roles (Smyth, 1998). It could also be due to the fact that men and women differ in how they construct narratives about significant experiences, with men taking a more problem-focused approach (Ptacek et al., 1994).

There are some limitations to consider in the present study. First, we chose to mainly focus on the identity-relevance in the writing instructions and not make specifications about any other aspects of the memory (e.g., valence or age), which could ultimately have influenced our coherence scores. For example, memories of very recent experiences might be more incoherent in general, because there have been less opportunities to mentally process this event and to talk about it to others. Especially for negative experiences, it might take a substantial amount of time and mental rehearsal to be able to make sense out of that experience and construct a coherent narrative about it. Second, because the study is correlational in nature, no conclusions can be drawn about the direction of the relationship between narrative coherence and identity. Experimental studies manipulating either coherence or sense of self could possibly shed light on their causality. Studies looking at longitudinal associations between coherence and identity could also be interesting. Third, the use of a community sample probably influences the strength of the associations. In general, coherence is already fairly high in a healthy sample. It is important that future studies also examine these associations in a clinical sample (e.g., patients with Borderline Personality Disorder), as this population tends to be more incoherent and identity functioning is more impaired. It is not unlikely that the predicted associations will be more pronounced if a clinical sample is included.

In conclusion, results indicate that narrative coherence and identity are related. For psychological well-being and internalizing symptoms, coherence was only significantly related with certain subcomponents. Within individuals, associations were not significantly stronger for memories with high identity-relevance compared to memories with low identity-relevance. However, we did observe gender differences indicating that although women construct more coherent accounts of their memories, it might be especially beneficial for men. Longitudinal and experimental studies are needed to further clarify how narrative coherence, identity, and well-being are related.

## Ethics Statement

This study was carried out in accordance with the recommendations of the Declaration of Helsinki and the European General Data Protection Regulation (GDPR). All subjects gave written informed consent in accordance with the Declaration of Helsinki. The protocol was approved by the Social and Societal Ethics Committee of the KU Leuven.

## Author Contributions

The data were collected, coded, and analyzed by LV, after frequent meetings with DH to brainstorm about the design. LV wrote the manuscript, but DH revised this manuscript on multiple occasions, providing valuable feedback.

### Conflict of Interest Statement

The authors declare that the research was conducted in the absence of any commercial or financial relationships that could be construed as a potential conflict of interest.

## Footnotes

^1^https://aspredicted.org/qk6tn.pdf

## APPENDIX A

**Example Narratives**

**Coherent narrative with high identity-relevance**

While I was attending college my grandfather died. I was shocked because he was still in good health the last time I visited him. He died from a seizure and I had to leave school for a couple of weeks to attend the funeral and check up on my family. It was all very somber and sad. My grandfather was really looking forward to me graduating since no one in my immediate family had done it. I was doing well in school, but my grades were nothing to brag about. I returned to college after a few weeks and thought about my grandfather’s death and I reflected on his life. After his death, I was motivated even more to graduate and over the course of the next semesters my grades significantly improved. I finished all of my school work before I went out with friends and I studied longer for exams and tests. My grandfather’s death has made me become a more serious person who is more focused on finishing what I start.

[context = 3; chronology = 3; theme = 3]

**Incoherent narrative with high identity-relevance**

I would say that this memory probably involved my time in high school. There were parts of high school that were more or less significant and more or less meaningful, but overall, it seemed to be both meaningful and impactful, though perhaps, the more meaningful parts were less impactful and vice versa. During this time, I was really able to express myself through creative enterprises and through social bonding, and felt like this was important to me, as I was often acting very authentically. On the other hand, I would engage in a lot of projects that were definitely impactful, and I felt like they were somehow more meaningful to me or better represented my core values because I felt like I was better able to personally express who I really was while engaging in them, relative to how I have often felt in the workplace. Obviously, there is not really a way to express one’s most crucial values through certain kinds of academic assessments, but, when I think back to presentations and projects I engaged in, relative to what I have accomplished in the workplace, it seems like I was more able to show who I was over the course of my academic career.

[context = 1; chronology = 0; theme = 0]

**Coherent narrative with low identity-relevance**

An important event in my life that had no impact on my sense of self was when my cat died earlier this year. This event impacted me greatly because I had the cat since I was in elementary school and had loved him so much. He was truly a member of the family. I found out that he had to be put down the day after it happened. I was on vacation, so I was unable to be there for it, which affected me very negatively. I felt sadness and regret and wished desperately that I could have been there for him. My sister called me the day after and told me everything that had occurred. He had begun coughing up blood, so they rushed him to the emergency vet. At the vet, they found out that he had cancer and that his organs had begun failing. The vet asked if they would like to take him home or have him put down, but my sister determined that the best option for his health and happiness was to put him down. It was an incredibly hard and heartbreaking decision to have to make, but I know it was the right one. I still miss him very much, and most likely always will.

[context = 3; chronology = 3; theme = 3]

**Incoherent narrative with low identity-relevance**

Funny enough, a lot of things like graduating can fit in here. I mean, I had a degree at a place that I’d always had great feelings about, and that was a great thing, right? I’d spent all this time getting a degree at the only place that I’d ever really considered as somewhere to go, and done a lot of interesting things and had more experiences that I really wasn’t thinking were something that I’d ever do, and I feel like I’m someone from there… but the actual memories of being there don’t really make that much of an impact. They’re all great things that happened, but none of them are really about my identity – all the things that happened there were gradual, and not something that I can easily point to, but I don’t really know that anything about my identity changed there, and so… I don’t really know, but while it has to have really shaped me, none of those memories feel like they’re all that central to my identity. I’m certainly not the same person that I was when all of this started, but I don’t feel like any of those memories really have a relation to my current self.

[context = 0; chronology = 0; theme = 0]
